# Mental Health in the digital area: what every doctor should know

**DOI:** 10.1192/j.eurpsy.2023.184

**Published:** 2023-07-19

**Authors:** R. M. Molina-Ruiz

**Affiliations:** Psychiatry, Hospital Clinic San Carlos, Madrid, Spain

## Abstract

**Abstract:**

The use of social networks is an integral part of our daily life as a means of communication. Patients manage information mostly from the internet, that strongly influence their beliefs and behaviors toward illness. Scientific dissemination through these kinds of platforms has expanded enormously in recent years, varying between them in style, contents and type of interaction with the users, that make necessary an individualized analysis. The use of Instagram goes beyond sharing photos and free comments, and it has been used extensively in different fields of medicine, such as mental health. This tool has enormous potential as a means of more effective communication and prevention and it is very valuable as a tool for psychoeducation and prevention in mental health. However, in the era where “information is easy to get but knowledge is difficult to find”, professionals of mental health should get involved and adapt to new scenarios of communication.

**Disclosure of Interest:**

None Declared

